# Supplementary material for: Expression of segment polarity genes in brachiopods supports a non-segmental ancestral role of engrailed for bilaterians
Source: Sci Rep. 2016 Aug 26;6:32387. doi: 10.1038/srep32387 (PMC4999882; doi:10.1038/srep32387)
Supplement: Supplementary Information [file srep32387-s1.pdf]

Supplementary Information

Expression of segment polarity genes in brachiopods supports a non-segmental ancestral role of *engrailed* for bilaterians

Bruno C. Vellutini[1] and Andreas Hejnol[2*]

[1,2]Sars International Centre for Marine Molecular Biology, University of Bergen, Thormøhlensgate 55, 5008 Bergen, Norway.

[1]Email: bruno.vellutini@uib.no

[2]Email: andreas.hejnol@uib.no

[*]Corresponding author.

Supplementary discussion

Candidate genes are not iterated through all four partitions of *N. anomala* mesoderm

Expression of *en* borders the posterior boundary of the second and third coelomic sacs in the mesoderm of *N. anomala*. These domains initiate adjacent to the preceding ectodermal domains of *en*, suggesting the ectoderm might signal to the mesoderm during brachiopod development. None of the other genes expressed in the mesoderm of *N. anomala* (*pax2/5/8*, *ptc*, *smo* and *gli*) show iterated patterns throughout the four coelomic sacs. In addition, *en*, *ptc*, *smo* and *gli*, but not *pax2/5/8*, are also expressed in the unsegmented mesoderm of *T. transversa*, suggesting that their developmental role might not be related to the partitioned mesoderm morphology of *N. anomala*.

The connection between the ectodermal and the mesodermal expression of *en* was also found in annelids[1], even though the expression patterns do not resemble that of brachiopods[1,2]. The mesodermal expression of *en* in *N. anomala* is similar to the more distantly related amphioxus[3] and onychophorans[4,5], where *en* transcripts are localized to the posterior portion of the mesodermal somites. However, the actual role of a posterior *en* domain in the mesoderm is unknown, and requires further investigation in these animals.

Over-activation of the Wnt pathway abolishes *en* and *wnt1* but not *pax6* expression at the apical/mantle boundary

Over-activation of the Wnt pathway caused an expansion of the pedicle lobe and of the domains of gene expression therein, suppressed the formation of the mantle lobe and of the apical/mantle furrow, and shifted other expression domains anteriorly. Embryos treated at the mid-blastula stage do not show morphological or molecular traces of a mantle lobe. We thus suggest the boundary between the anterior and posterior portions of treated larvae does not correspond to any of the wild type boundaries, but to a unique apical/pedicle boundary defined by an ectodermal fold. Embryos treated at later stages (radial gastrula) show bilateral stubs of mantle tissue, suggesting

the specification of the mantle lobe of *T. transversa* might initiate between mid-blastula and radial gastrula stage. The period between mid-blastula and early gastrulation can also play a role in the positioning of the anterior domains of *en* and *wnt1*—but not in the establishment of *pax6* expression. Disturbance of *en* and *wnt1* expression in the mid-blastula treatments suggests these anterior domains are not yet established in the embryo, and their positioning is affected by the over-activation of the Wnt pathway. Differently, the consistent expression of *pax6* between mid-blastula and radial gastrula treatments indicates the anterior domain of *pax6* was not affected by the azakenpaullone, suggesting its specification must occur before the mid-blastula stage or be independent of the Wnt pathway. Thus, our experimental data suggests that disturbing *T. transversa* Wnt signaling affects the development of the mantle lobe and the morphology of the apical/mantle boundary, and that *pax6* might have an earlier upstream role in the embryonic body patterning of brachiopods.

Supplementary figures

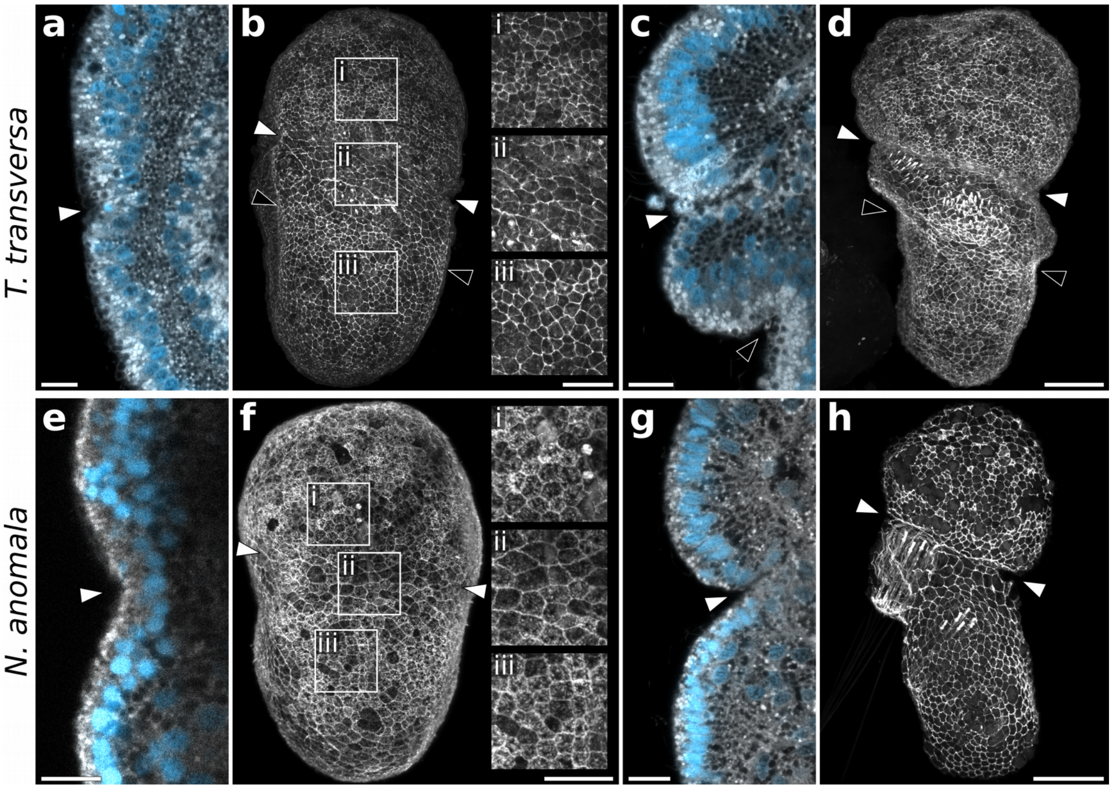

Supplementary Figure S1. Morphology of the ectodermal boundaries in the larvae of the brachiopods *T. transversa* and *N. anomala*. (a,e) Outline of the apical/mantle furrow in the epidermis of the bilateral gastrula in ventral view. (b,f) Lateral view of the bilateral gastrula epidermal surface showing the membrane outlines of epithelial cells. Cells abutting the posterior border of the furrow are more elongated compared to the isodiametric shape of anterior and posterior cell populations. Boxes mark the regions corresponding to the insets on the right side (i–iii). (c,g) Outline of the apical/mantle furrow in the epidermis of the bilobed larva in ventral view. (d,h) Lateral view showing the epidermal surface of the bilobed larva showing the membrane outlines of epithelial cells. Nuclei stained with DAPI (blue) and cytoplasmic staining (gray) obtained by mounting specimens stained with BODIPY FL in Thiodiethanol (a,c,e,g). Cell membrane outlines (F-Actin) stained with BODIPY FL mounted in Murray's Clear (b,d,f,h). White arrowheads mark the apical/mantle boundary and black arrowheads mark the mantle/pedicle boundary. Anterior is top and ventral is to the right in lateral views for all panels. Scale bars = 20 µm (b,d,f,h), 10 µm (a,c,e,g).

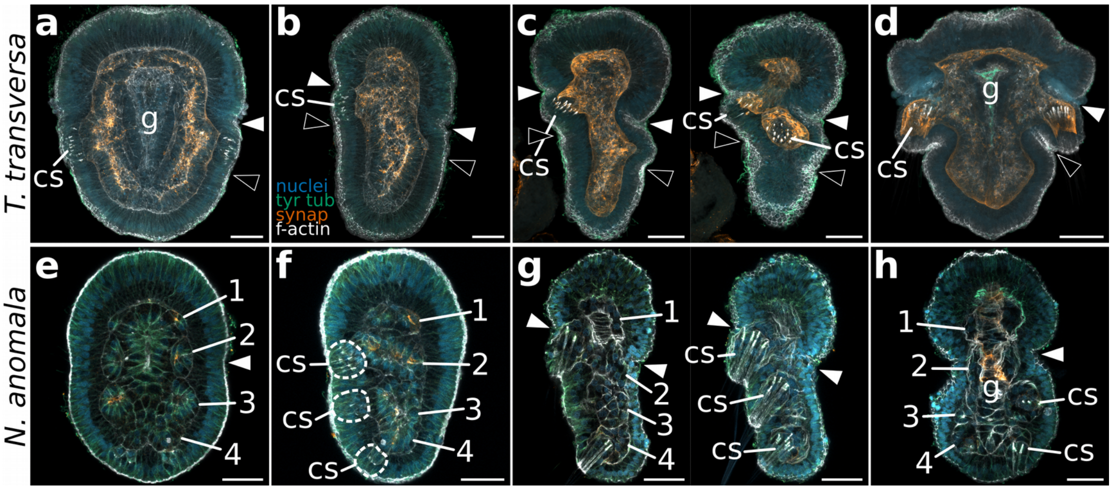

Supplementary Figure S2. Morphology of the mesodermal boundaries in the larvae of the brachiopods *T. transversa* and *N. anomala*. (a,e) Mesoderm morphology at the bilateral gastrula in a ventral view. While *T. transversa* mesoderm has no divisions (a), *N. anomala* mesoderm has lateral constrictions individualizing each of the four pairs of coelomic sacs (e). (b,f) Same stage, but lateral view, revealing that *N. anomala* mesoderm remains interconnected more medially in the ventral side (f). (c,g) Bilobed larva showing a more medial (left) and a more lateral (right) optical sections of an embryo in lateral view. Mesoderm is associated to the developing chaetae sacs (cs). (d) Ventral view of a *T. transversa* trilobed larva showing its unsegmented mesoderm with a distinct anterior portion in the apical lobe, an umbrella-like mesoderm in the mantle lobe connected to the chaetae sacs and an arrowhead-shaped pedicle portion. (h) Ventral view of a developed bilobed larva of *N. anomala* with reduced coelomic spaces. Mesoderm evidenced by the antibody Anti-Synapsin II (vermillion) with the counter staining of tyrosinated tubulin (green), F-Actin (white) and DAPI (blue). Anti-Synapsin II cross-react with an unknown component of *T. transversa* mesoderm and was used as a marker; this reaction is weaker and less specific in *N. anomala*. The four coelomic sacs of *N. anomala* are indicated by numerals 1–4 from anterior to posterior. cs: chaetae sac, g: gut. White arrowheads mark the apical/mantle boundary and black arrowheads mark the mantle/pedicle boundary. Anterior is top and ventral is to the right in lateral views for all panels. Scale bars = 20 µm.

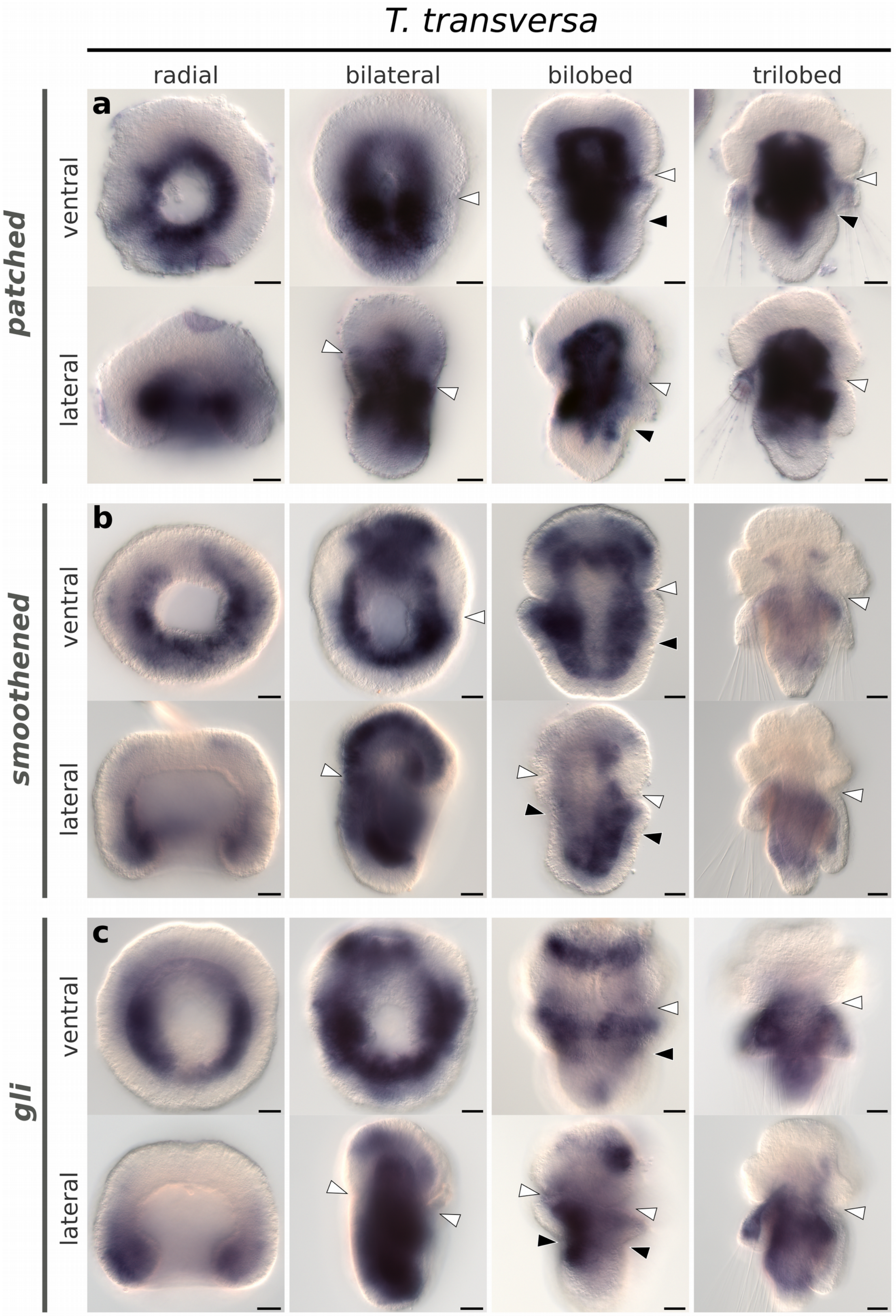

Supplementary Figure S3. Whole mount in situ hybridization of the Hedgehog pathway orthologs in representative developmental stages of the brachiopod *T. transversa*. (a) Expression of *ptc*. (b) Expression of *smo*. (c) Expression of *gli*. In the radial gastrula, transcripts of *ptc*, *smo* and *gli* occur in the mesoderm. Transcripts of *ptc* are expressed in the mesoderm and endoderm of subsequent stages, except at the posterior pedicle mesoderm. The gene *smo* is ubiquitously expressed in the mesoderm dur-

ing gastrulation and, in the asymmetric and bilateral gastrula, in a conspicuous anterior ectodermal domain. Expression of *smo* cleared from the anterior mesoderm in the trilobed larva. Expression of *gli* covers the whole mesoderm of the radial and asymmetric gastrula, and in two additional ectodermal domains at the anterior region of the apical lobe and mantle lobe. Expression becomes restricted to patches in the mantle mesoderm and the ectodermal domains resolve into stripes in the anterior portion of the apical and mantle lobes in the bilobed larva. Finally, in the trilobed larva transcripts of *gli* are restricted to the mantle and pedicle mesoderm. Anterior is top in all panels and ventral is to the right in all lateral views. White arrowheads mark the apical/mantle boundary and black arrowheads mark the mantle/pedicle boundary. Scale bars = 20 µm.

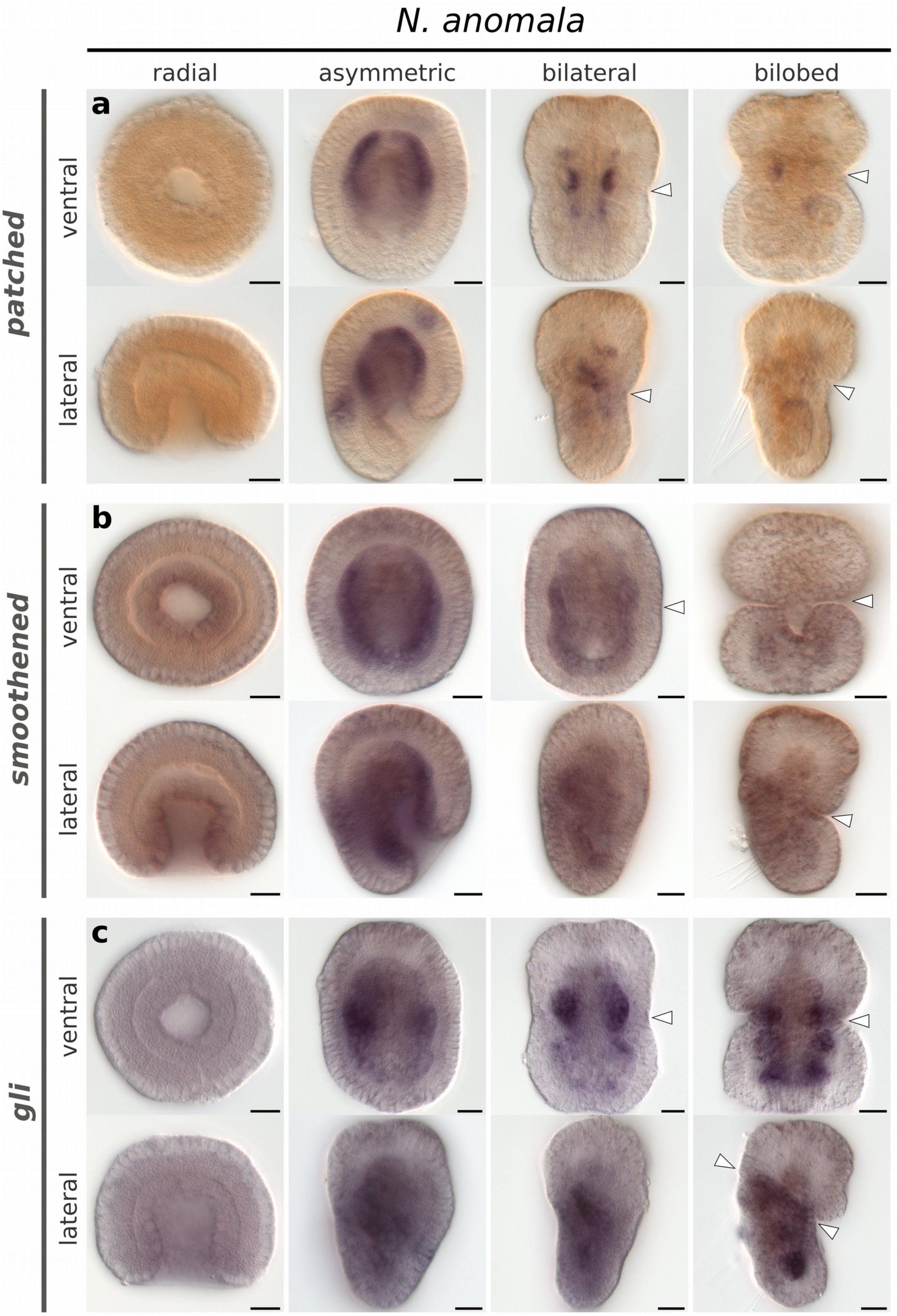

Supplementary Figure S4. Whole mount in situ hybridization of the Hedgehog pathway orthologs in representative developmental stages of the brachiopod *N. anomala*. (a) Expression of *ptc1*. (b) Expression of *smo*. (c) Expression of *gli*. In the radial gastrula, *ptc1* is not expressed, whereas in the asymmetric gastrula it is restricted to the anterior mesoderm and anterior-dorsal domains. Transcripts of *ptc1* remain in the ventral mesoderm associated with the first, second and third coelomic sacs and fade in the

bilobed larva. The expression of *smo* in the asymmetric gastrula covers most of the mesoderm except for the anterior portion correspondent to the first coelomic sac; a dorsal ectodermal domain is also present. The expression fades in the bilateral gastrula and *smo* is not detected in the bilobed larva. The gene *gli* is expressed in the forming second to fourth coelomic pouches, but not the in the first, and the expression covers most of the mesoderm in the bilobed larva. Anterior is top in all panels and ventral is to the right in all lateral views. White arrowheads mark the apical/mantle boundary. Scale bars = 20 µm.

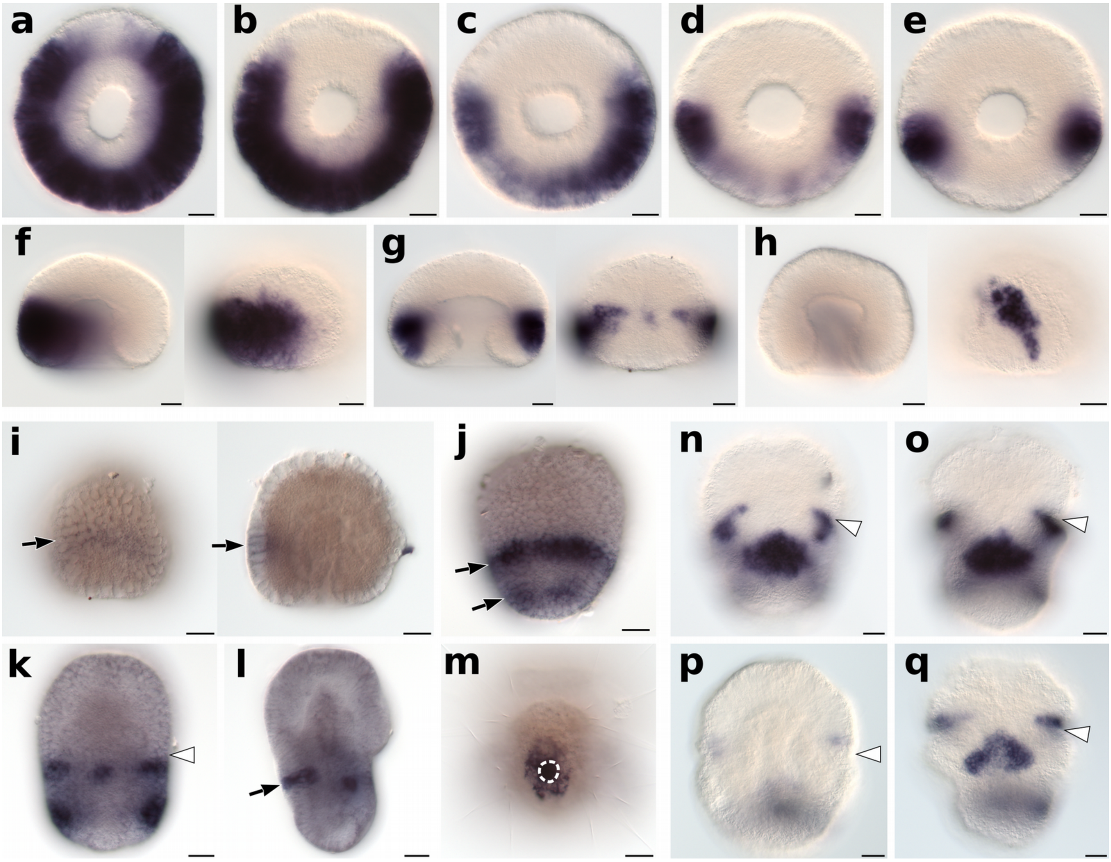

Supplementary Figure S5. Additional details about the embryonic expression of *en* and *wnt1* in the brachiopods *T. transversa* and *N. anomala*. (a–e) Dynamic progression of *en* transcripts at the radial gastrula of *T. transversa* in blastoporal view. (f,g,h) Lateral views correspondent to (b,d,e), respectively, with anterior to the right side. (i) Dorsal (left) and lateral view (right) of *N. anomala* radial gastrula showing faint *en* stripe (arrow). (j) Dorsal view of *N. anomala* asymmetric gastrula with two stripes of *en* (arrows). (k) Dorsal view of *N. anomala* bilateral gastrula. (l) Conspicuous dorsal domain of *en* associated with the shell rudiment in the bilobed larva of *N. anomala* in lateral view (arrow). (m) Developed larva of *N. anomala* with shell primordium. Original area with *en* signal is outlined by white dashed line. Surrounding area is likely unspecific staining related to the shell deposition. (n) Dorsal view of a *T. transversa* bilateral larva showing *en* expression. (o) Same view as (n) showing *en* expression at the subsequent bilobed larva stage. (p) Absence of *wnt1* expression on the dorsal side of *T. transversa* bilateral gastrula. (q) Same view as (p) showing dorsal *wnt1* domain of *T. transversa* bilobed larva. Scale bars = 20 µm.

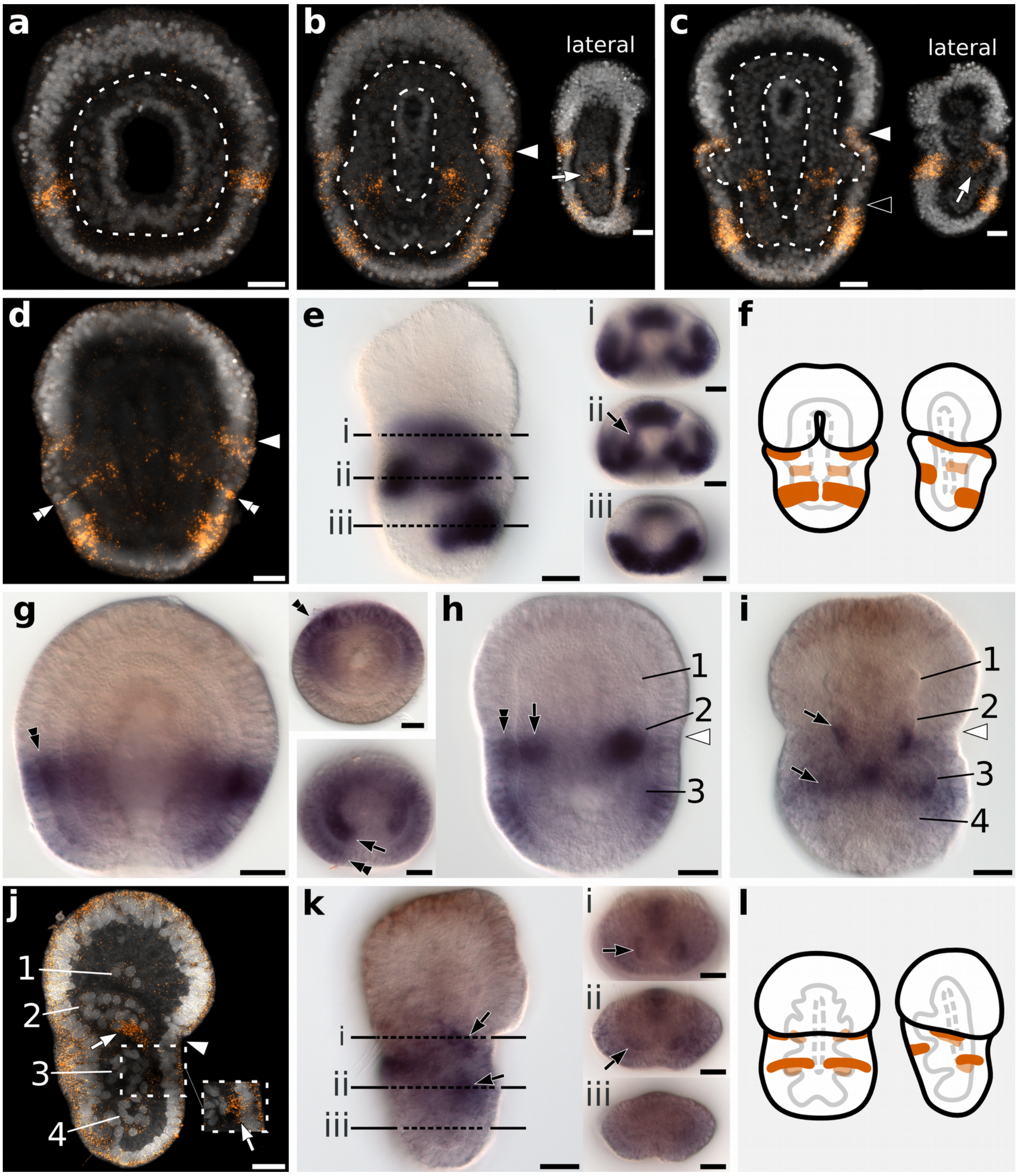

Supplementary Figure S6. Whole mount in situ hybridization *en* orthologs in representative developmental stages of the brachiopods *T. transversa* and *N. anomala*. Anterior is top in all panels and ventral is to the right in all lateral views. (a–f) Expression of *en* in the mesoderm of *T. transversa*. White dashed lines are highlighting the mesoderm. (a) Asymmetric gastrula with no mesodermal expression of *en*. (b) Onset of mesodermal *en* in the bilateral gastrula in a ventral and lateral view (inset). Specimens are the same of Figure 2c. (c) Resolved bands of *en* in the mesoderm of the bilobed larva in a ventral and lateral view (inset). (d) Connection between ectoderm and mesoderm cells expressing *en* in a bilateral gastrula. (e) Lateral view of bilobed larva. Black dashed lines mark the position of the optical sections of a posterior view of the same embryo (insets i–iii). (f) Line drawings represent expression at the bilobed stage of *T. transversa*. (g–l) Expression of *en* in the mesoderm of *N. anomala*. (g) Asymmetric gastrula with weak mesodermal staining of *en* in a ventral and posterior view (inset). (h) Strong *en* domains in the mesoderm of the bilateral gastrula adjacent to the ectodermal domains at the posterior region of coelomic sac “2”. Inset shows the mesoderm in a posterior view. (i) Two pairs of mesodermal domains of *en* in the pos-

terior portion of the coeloms “2” and “3” of the bilobed larva. (j) Detail of *en* expression in the bilobed larva in the coeloms “2” and “3” (inset). (k) Lateral view and optical sections of a posterior view of a bilobed larva showing the expression in the coelomic sacs (insets). (l) Line drawings represent expression at the bilobed stage of *N. anomala*. White arrowheads mark the apical/mantle boundary and black arrowheads mark the mantle/pedicle boundary. Double arrowheads mark the ectodermal expression of *en* while arrows point to the mesodermal domains. The four coelomic sacs of *N. anomala* are indicated by numerals 1–4 from anterior to posterior. Scale bars = 20 µm.

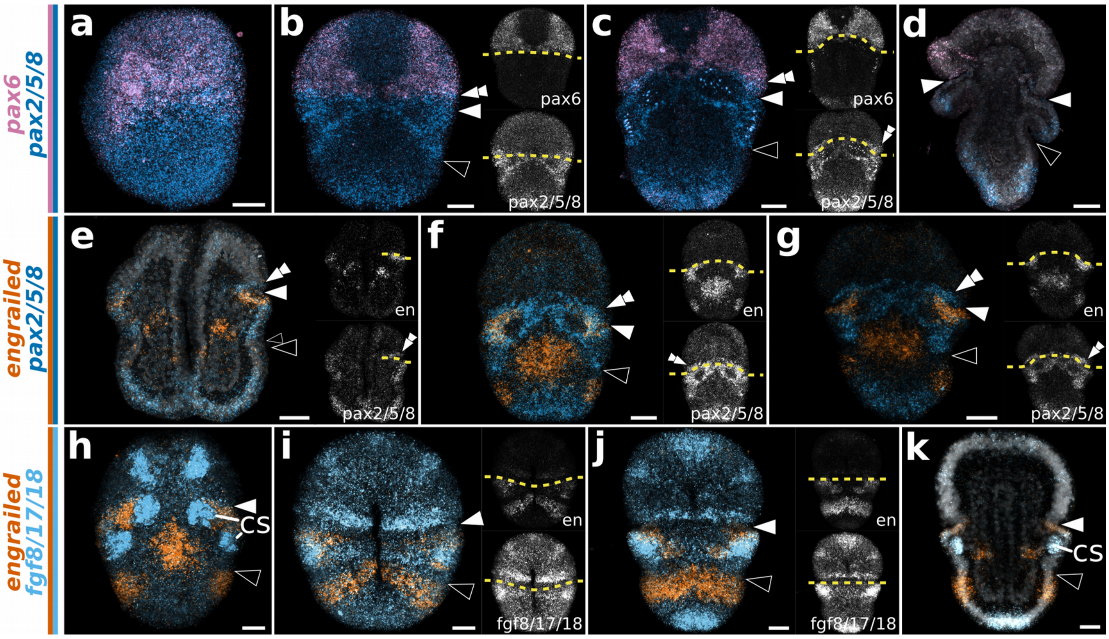

Supplementary Figure S7. Whole mount double fluorescent in situ hybridization of *pax6* + *pax2/5/8* (a–d), *en* + *pax2/5/8* (e–g) and *en* + *fgf8/17/18* (h–k) in the brachiopod *T. transversa*. (a) Radial gastrula with overlapping domains of *pax6* and *pax2/5/8*. (b,c) Dorsals views of a bilateral and a bilobed stage highlighting that the posterior limit of *pax6* expression borders the apical/mantle boundary while the *pax2/5/8* domain crosses the furrow anteriorly. (d) Trilobed larva. (e) Detail of the apical/mantle boundary. (f,g) Dorsal views of a bilateral and a bilobed stage showing the expression of *en* and *pax2/5/8*. (h) Asymmetric gastrula exhibiting *fgf8/17/18* expression in the chaetae sac primordia (cs) with non-overlapping *en* domains. (i,j) Ventral views of a bilateral and a bilobed stage highlighting that *fgf8/17/18* does not directly border the apical/mantle boundary. (k) Section of (j) showing the *fgf8/17/18* expression in the chaetae sacs (cs). Anterior is top in all panels and ventral is to the right in all lateral views. Side panels show the relation between gene expression and the apical/mantle boundary (striped yellow line). White arrowheads mark the apical/mantle boundary and black arrowheads mark the mantle/pedicle boundary. Scale bars = 20 µm.

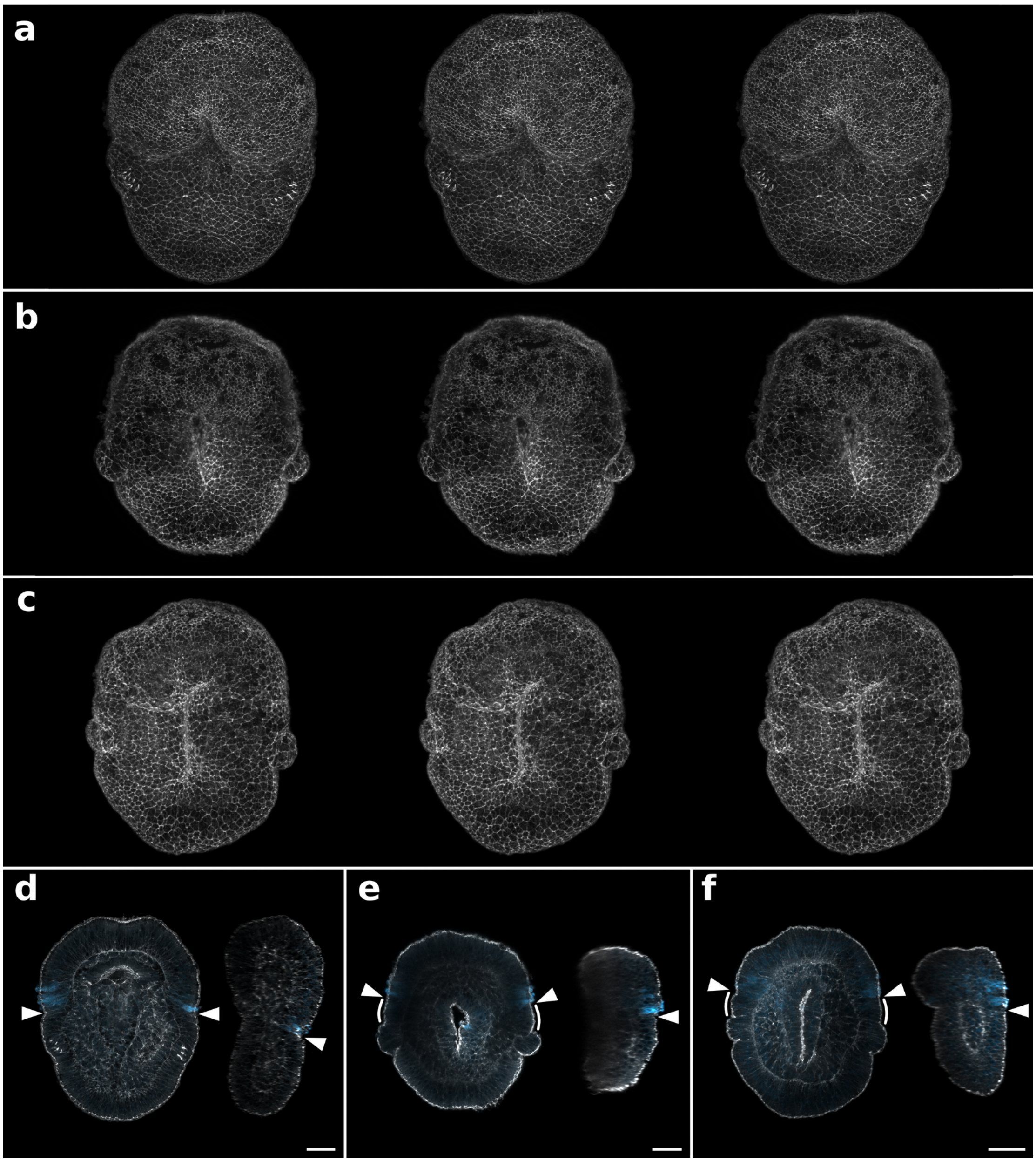

Supplementary Figure S8. Perturbation of the apical/mantle boundary during over-activation of Wnt pathway in *T. transversa*. (a–c) Stereo images showing a maximum intensity projection of the epithelial surface of embryos stained for F-actin (cell membranes in white). (d–f) Maximum intensity projections of five inner optical sections (left) and orthogonal views (right) of the same embryos as above, but including nuclei staining (blue) to highlight the position of corpuscular bodies. Control sample incubated with DMSO shows the wild type ectodermal furrow at the apical/mantle boundary (a,d). Embryos treated with 1 µM (b,e) and 10 µM (c,f) 1-azakenpaullone do not form a furrow at the apical/mantle boundary. Arrowheads mark the posterior end of the apical lobe. White line marks the area between the expected furrow position and the mantle lobe stub in treated embryos (E, F). Scale bars = 20 µm.

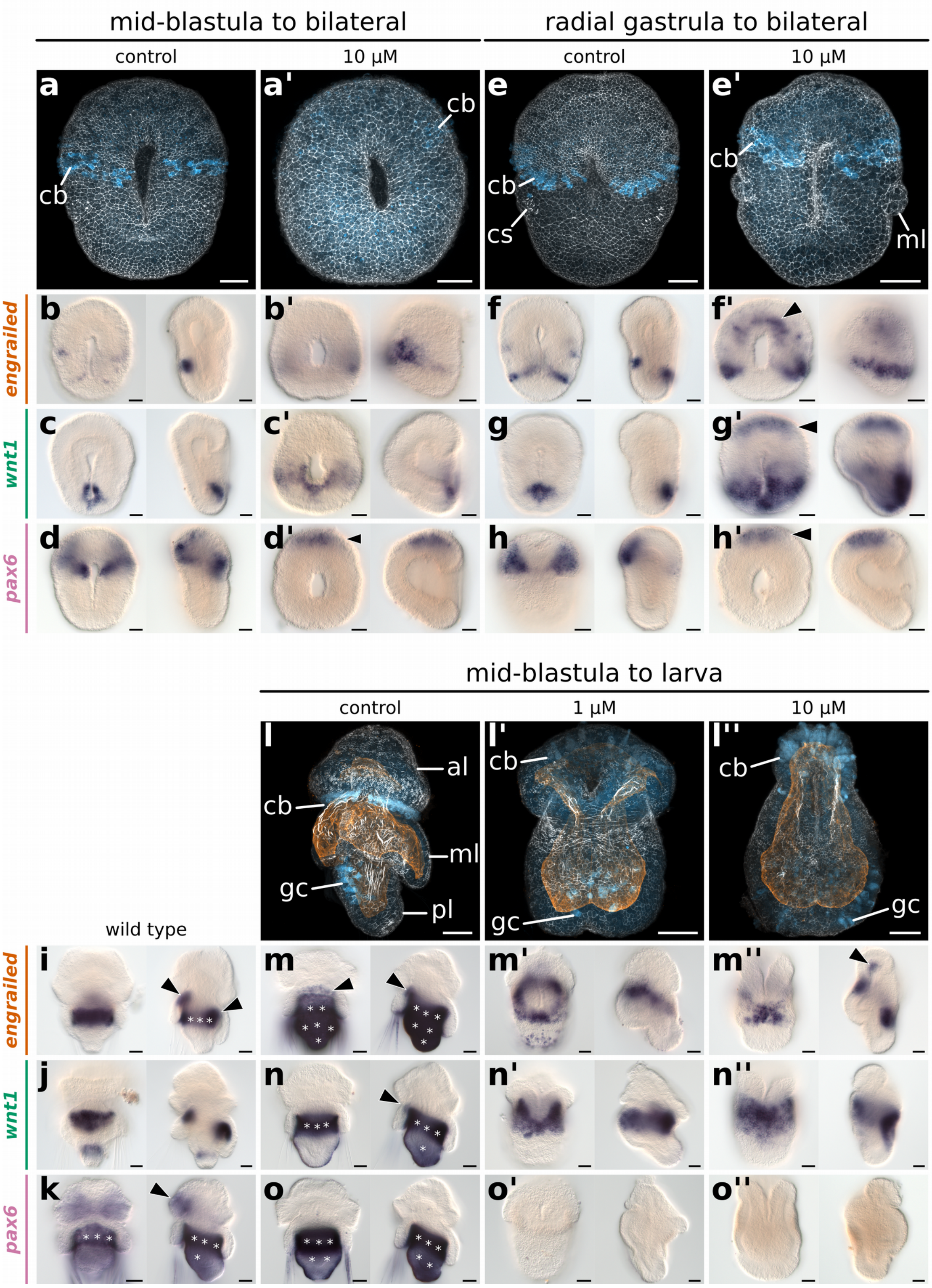

Supplementary Figure S9. Over-activation of the canonical Wnt pathway on the development of the brachiopod *T. transversa*. We treated developing embryos of *T. transversa* with 1 and 10 µM 1-azakenpaullone from the mid-blastula to bilateral gastrula stage (a–d), radial gastrula to bilateral gastrula stage (e–h) and mid-blastula to trilobed larval stage (l–o). (a,a') Control and treated samples from mid-blastula to bilateral gastrula. Embryos did not elongate and the organization of the corpuscular bodies (cb)—cells of unknown function that protrude out of the epithelium—is disrupted. (e,e') Control and treated samples from radial gastrula to bilateral gastrula. The apical/mantle furrow is not well defined and the embryo shows bilateral stubs

correspondent to incipient mantle lobe tissue. Treated embryos show no sign of chaetae formation. (b,b',f,f') Expression of *en* in control and treated embryos. Control embryos have an apical/mantle stripe, a ventral and a dorsal domain of *en*. Early (mid-blastula) and late (radial gastrula) treatments show a posterior laterodorsal *en* domain, but only in the latter we detected an anterior *en* domain consisting of scattered ectodermal cells, and a broad mesodermal domain in the anterior end (arrowhead). (c,c',g,g') Expression of *wnt1* in control and treated embryos. Control embryos have *wnt1* expressed around the posterior lip of the blastopore. In the treated samples there is an expansion of this posterior domain, occupying half of the blastopore length. Only embryos treated at the radial gastrula stage show an anterior expression domain of *wnt1* at the apical plate (arrowhead), separated from the posterior domain by a subterminal band clear of expression. (d,d',h,h') Expression of *pax6* in control and treated embryos. Control embryos have *pax6* transcripts contained in the apical lobe. In all treated embryos the expression of *pax6* shifts anteriorly localizing to the apical plate (arrowheads). (l) Morphology of control larvae incubated with DMSO. The apical lobe has a row of corpuscular bodies at its posterior portion, just above the apical/mantle furrow. The mantle lobe has outgrown with the extensive underlying mesoderm and musculature, and the pedicle lobe shows dorsal putative gland cells (gc). (l',l'') Morphology of the treated embryos reveals an expansion of posterior structures. (l') The larval body has a distinct anterior portion covered by corpuscular bodies and an unclosed blastoporal opening at its center. (l'') Higher concentrations of 1-azakenpaullone (10 µM) resulted in a diminished anterior portion and the morphology of the posterior portion resembles an over-developed pedicle lobe with putative gland cells on the dorsal side. An ectodermal fold divides the anterior and posterior regions and there were neither chaetae sacs nor evidence of a mantle lobe. (i,m,m',m'') Expression of *en* in wild type, control and treated embryos. (i) Wild type expression of *en* consists of a ventral and a dorsal domain in the pedicle lobe (arrowheads; same specimen as in Figure 3). (m) Control embryos show the same pattern, although regions of unspecific staining in the pedicle lobe (*) obfuscate the signal. (m',m'') Treated embryos show a broader expression domain of *en* encircling the embryo at the anterior portion of the pedicle lobe, similar to wild type and controls. At 10 µM, *en* is also expressed in the mesoderm at the anterior end (arrowhead). (j,n,n',n'') Expression of *wnt1* in wild type, control and treated embryos. (j) Wild type expression of *wnt1* colocalizes with *en* expression, except for an additional posterior domain at the pedicle lobe (same specimen as in Figure 3). (n) Control embryos display shell background (*), but signal at the dorsal side is visible (arrowhead). (n',n'') Transcripts of *wnt1* in treated embryos occupy the same territories as *en*, except for the absence of *wnt1* in the anterior mesoderm. (k,o,o',o'') Expression of *pax6* in wild type, control and treated embryos. (k) Wild type expression of *pax6* occurs in the dorsal portion of the apical lobe (arrowhead). We did not detect transcripts of *pax6* in control or treated embryos (o,o',o''). Scores represent the number of embryos with the phenotype shown in the panel against the total number of embryos analyzed: (b) 34/48, (b') 36/41, (c) 25/26, (c') 27/33, (d) 25/26, (d') 13/18, (f) 28/36, (f') 58/88 (21/88 without anterior mesodermal domain), (g) 56/59, (g') 57/72 (12/72 without anterior domain), (h) 28/31, (h') 51/53, (m) 30/33, (m') 38/43, (m'') 47/66 (11/66 without anterior mesodermal domain), (n) 13/24, (n') 34/38, (n'') 37/41, (o) 24/24, (o') 36/36, (o'') 29/29. Asterisks (*) mark unspecific staining due to the secretion of the larval shell in *T. transversa*. cb: corpuscular bodies, gc: putative gland cells, al: apical lobe, ml: mantle lobe, pl: pedicle lobe, cs: chaetae sacs. Scale bars = 20 µm.

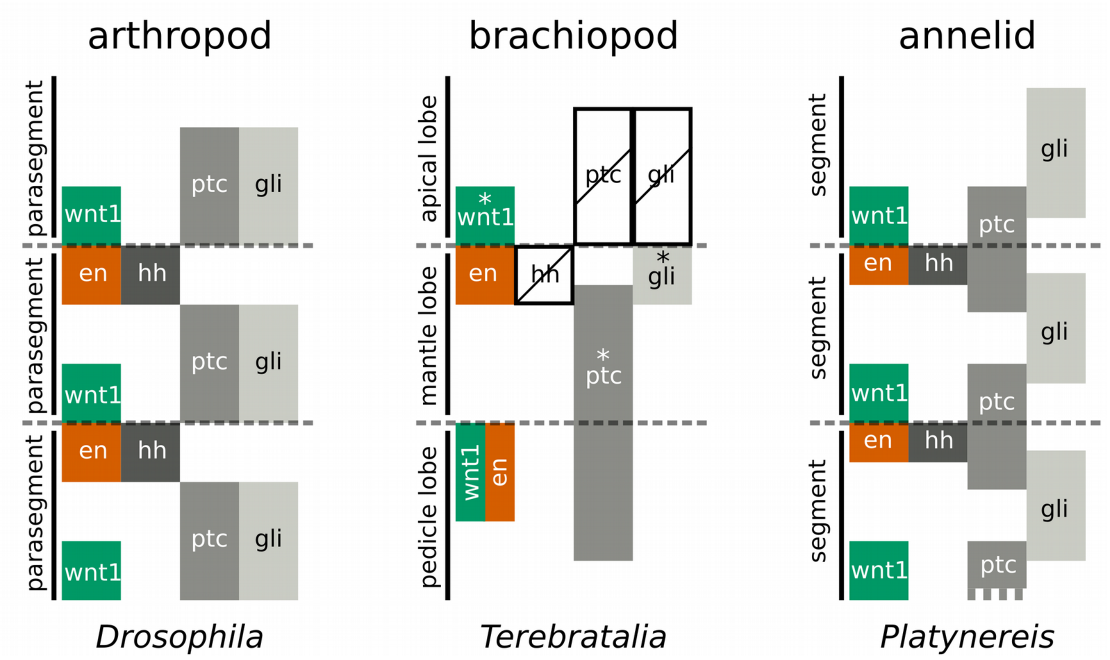

Supplementary Figure S10. Comparative expression of the segment polarity genes and components of the Hedgehog pathway. Expression patterns for *en*, *wnt1*, *hh*, *ptc* and *gli* in relation to the parasegments of an arthropod (*D. melanogaster*), the larval lobes of a brachiopod (*T. transversa*) and the body segments of an annelid (*P. dumerilii*). White boxes with black border indicate the expected localization of transcripts, if a segment polarity role was occurring in *T. transversa*. Asterisks mark expression patterns that are variable between *T. transversa* and *N. anomala*.

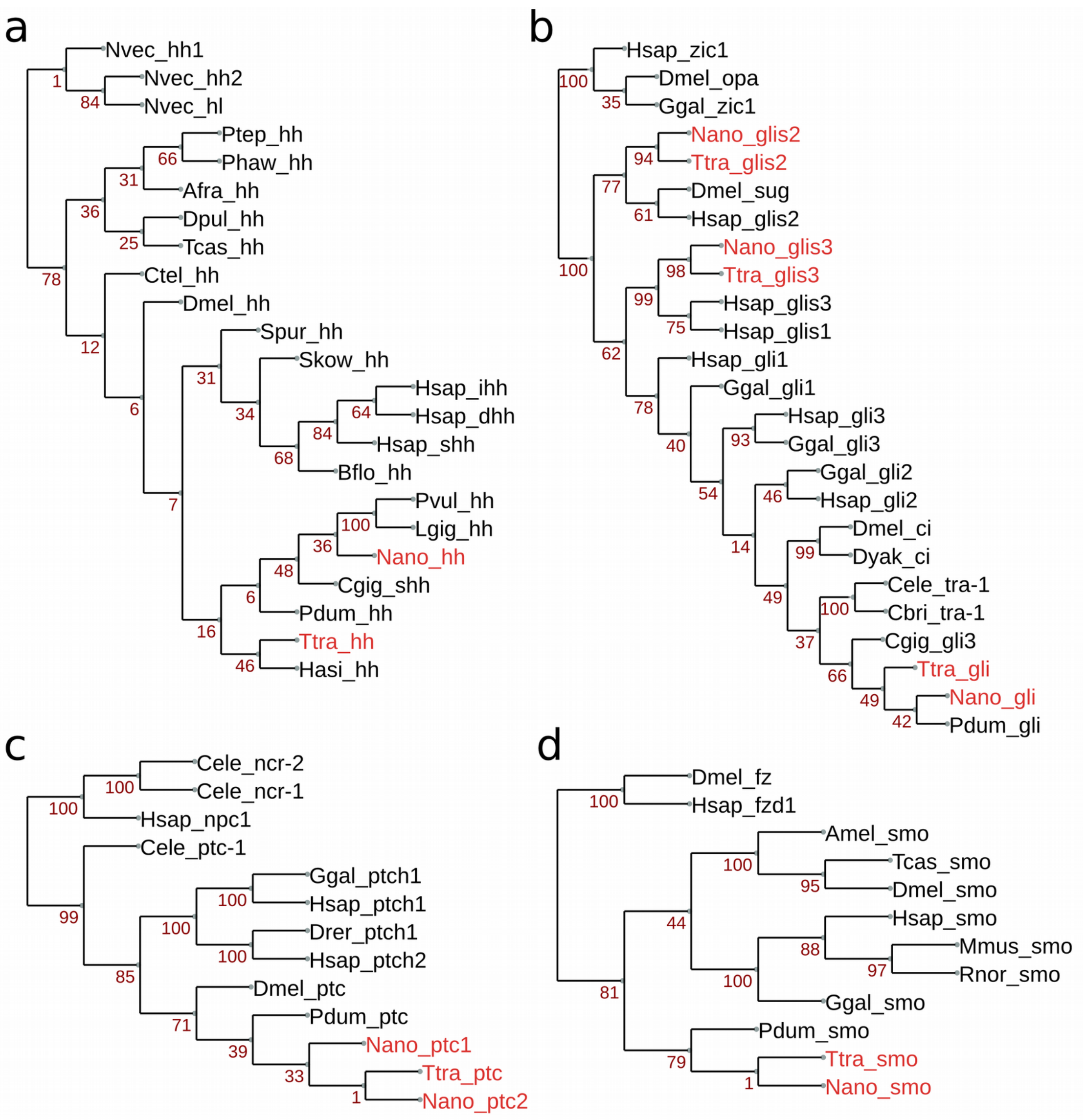
a
Nvec_hh1
Nvec_hh2
Nvec_hl
Ptep_hh
Phaw_hh
Afra_hh
Dpul_hh
Tcas_hh
Ctel_hh
Dmel_hh
Spur_hh
Skow_hh
Hsap_ihh
Hsap_dhh
Hsap_shh
Bflo_hh
Pvul_hh
Lgig_hh
Nano_hh
Cgig_shh
Pdum_hh
Ttra_hh
Hasi_hh
1
84
66
31
36
25
78
12
6
31
34
64
84
68
7
100
36
48
6
16
46
b
Hsap_zic1
Dmel_opa
Ggal_zic1
Nano_glis2
Ttra_glis2
Dmel_sug
Hsap_glis2
Nano_glis3
Ttra_glis3
Hsap_glis3
Hsap_glis1
Hsap_gli1
Ggal_gli1
Hsap_gli3
Ggal_gli3
Ggal_gli2
Hsap_gli2
Dmel_ci
Dyak_ci
Cele_tra-1
Cbri_tra-1
Cgig_gli3
Ttra_gli
Nano_gli
Pdum_gli
100
35
94
77
61
100
98
99
75
62
78
40
93
54
46
14
99
49
100
37
66
49
42
c
Cele_ncr-2
Cele_ncr-1
Hsap_npc1
Cele_ptc-1
Ggal_ptch1
Hsap_ptch1
Drer_ptch1
Hsap_ptch2
Dmel_ptc
Pdum_ptc
Nano_ptc1
Ttra_ptc
Nano_ptc2
100
100
99
100
100
100
85
71
39
33
1
d
Dmel_fz
Hsap_fzd1
Amel_smo
Tcas_smo
Dmel_smo
Hsap_smo
Mmus_smo
Rnor_smo
Ggal_smo
Pdum_smo
Ttra_smo
Nano_smo
100
100
95
44
88
97
100
81
79
1

e

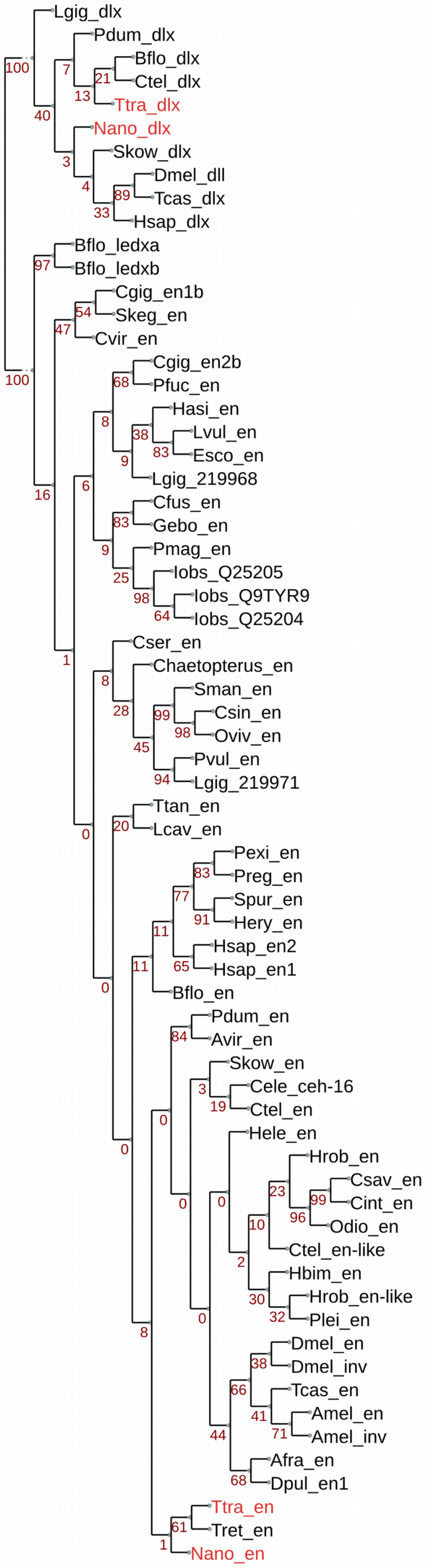
Lgig_dlx
Pdum_dlx
Bflo_dlx
Ctel_dlx
Ttra_dlx
Nano_dlx
Skow_dlx
Dmel_dll
Tcas_dlx
Hsap_dlx
Bflo_ledxa
Bflo_ledxb
Cgig_en1b
Skeg_en
Cvir_en
Cgig_en2b
Pfuc_en
Hasi_en
Lvul_en
Esco_en
Lgig_219968
Cfus_en
Gebo_en
Pmag_en
Iobs_Q25205
Iobs_Q9TYR9
Iobs_Q25204
Cser_en
Chaetopterus_en
Sman_en
Csin_en
Oviv_en
Pvul_en
Lgig_219971
Ttan_en
Lcav_en
Pexi_en
Preg_en
Spur_en
Hery_en
Hsap_en2
Hsap_en1
Bflo_en
Pdum_en
Avir_en
Skow_en
Cele_ceh-16
Ctel_en
Hele_en
Hrob_en
Csav_en
Cint_en
Odio_en
Ctel_en-like
Hbim_en
Hrob_en-like
Plei_en
Dmel_en
Dmel_inv
Tcas_en
Amel_en
Amel_inv
Afra_en
Dpul_en1
Ttra_en
Tret_en
Nano_en

f

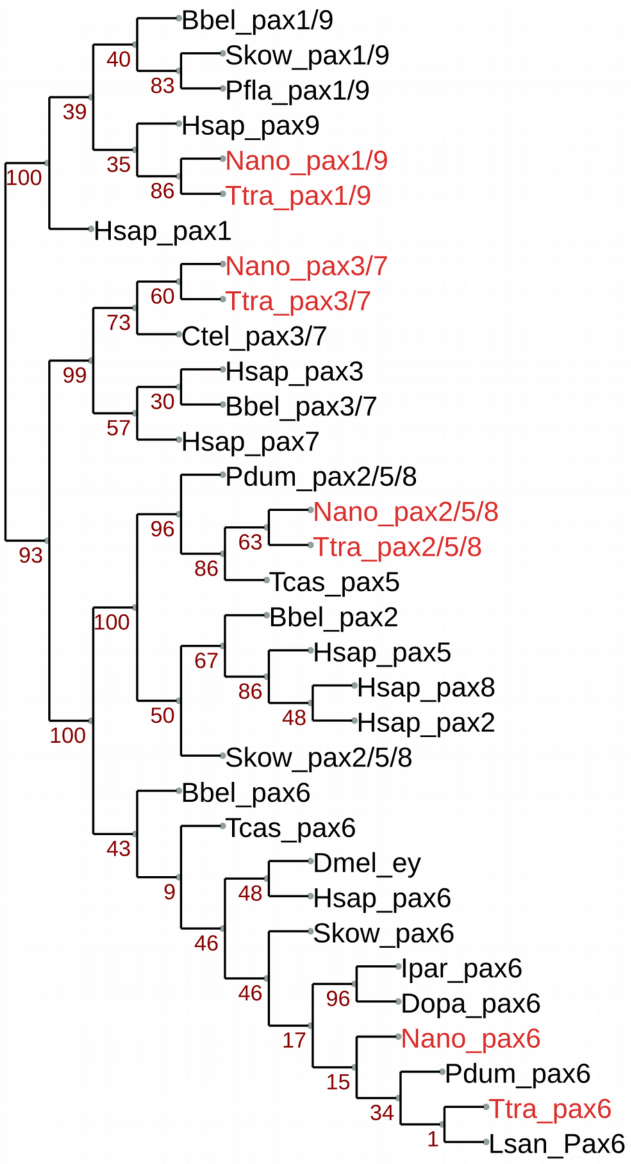
Bbel_pax1/9
Skow_pax1/9
Pfla_pax1/9
Hsap_pax9
Nano_pax1/9
Ttra_pax1/9
Hsap_pax1
Nano_pax3/7
Ttra_pax3/7
Ctel_pax3/7
Hsap_pax3
Bbel_pax3/7
Hsap_pax7
Pdum_pax2/5/8
Nano_pax2/5/8
Ttra_pax2/5/8
Tcas_pax5
Bbel_pax2
Hsap_pax5
Hsap_pax8
Hsap_pax2
Skow_pax2/5/8
Bbel_pax6
Tcas_pax6
Dmel_ey
Hsap_pax6
Skow_pax6
Ipar_pax6
Dopa_pax6
Nano_pax6
Pdum_pax6
Ttra_pax6
Lsan_Pax6
40
83
39
35
86
100
60
73
99
30
57
93
96
63
86
100
67
86
48
50
100
43
9
48
46
46
96
17
15
34
1

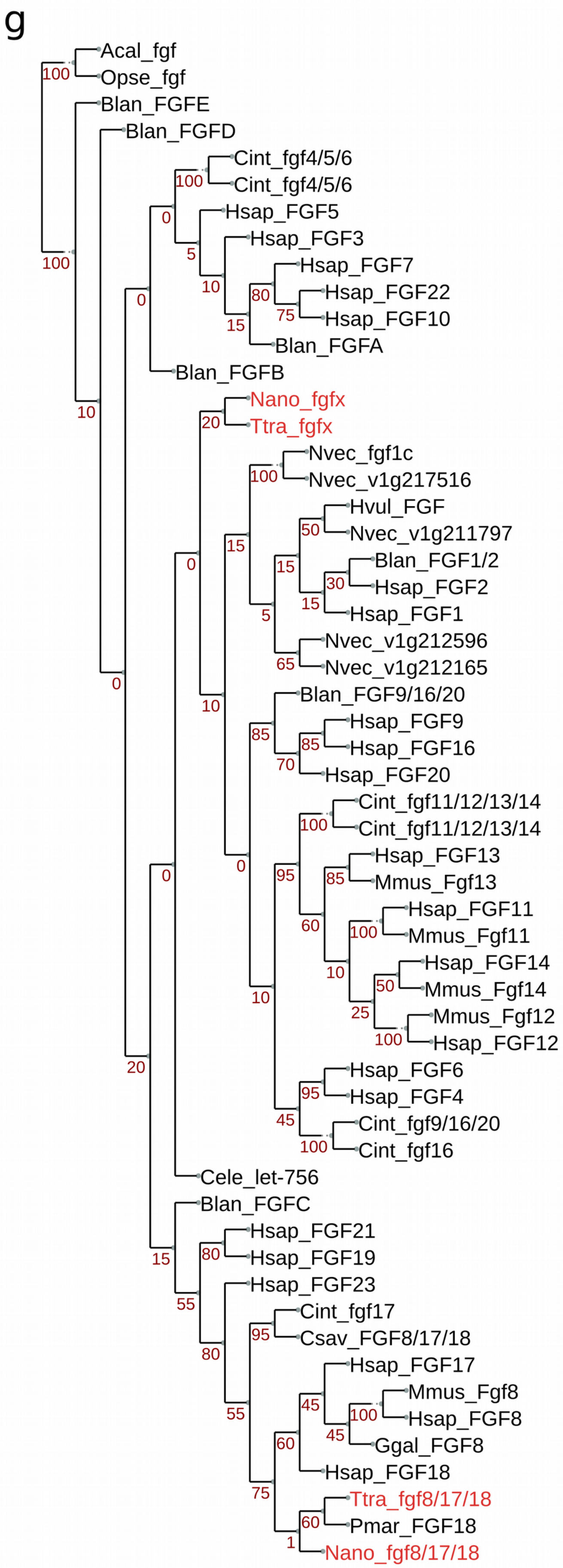
g
Acal_fgf
Opse_fgf
Blan_FGFE
Blan_FGFD
Cint_fgf4/5/6
Cint_fgf4/5/6
Hsap_FGF5
Hsap_FGF3
Hsap_FGF7
Hsap_FGF22
Hsap_FGF10
Blan_FGFA
Blan_FGFB
Nano_fgfx
Ttra_fgfx
Nvec_fgf1c
Nvec_v1g217516
Hvul_FGF
Nvec_v1g211797
Blan_FGF1/2
Hsap_FGF2
Hsap_FGF1
Nvec_v1g212596
Nvec_v1g212165
Blan_FGF9/16/20
Hsap_FGF9
Hsap_FGF16
Hsap_FGF20
Cint_fgf11/12/13/14
Cint_fgf11/12/13/14
Hsap_FGF13
Mmus_Fgf13
Hsap_FGF11
Mmus_Fgf11
Hsap_FGF14
Mmus_Fgf14
Mmus_Fgf12
Hsap_FGF12
Hsap_FGF6
Hsap_FGF4
Cint_fgf9/16/20
Cint_fgf16
Cele_let-756
Blan_FGFC
Hsap_FGF21
Hsap_FGF19
Hsap_FGF23
Cint_fgf17
Csav_FGF8/17/18
Hsap_FGF17
Mmus_Fgf8
Hsap_FGF8
Ggal_FGF8
Hsap_FGF18
Ttra_fgf8/17/18
Pmar_FGF18
Nano_fgf8/17/18

h

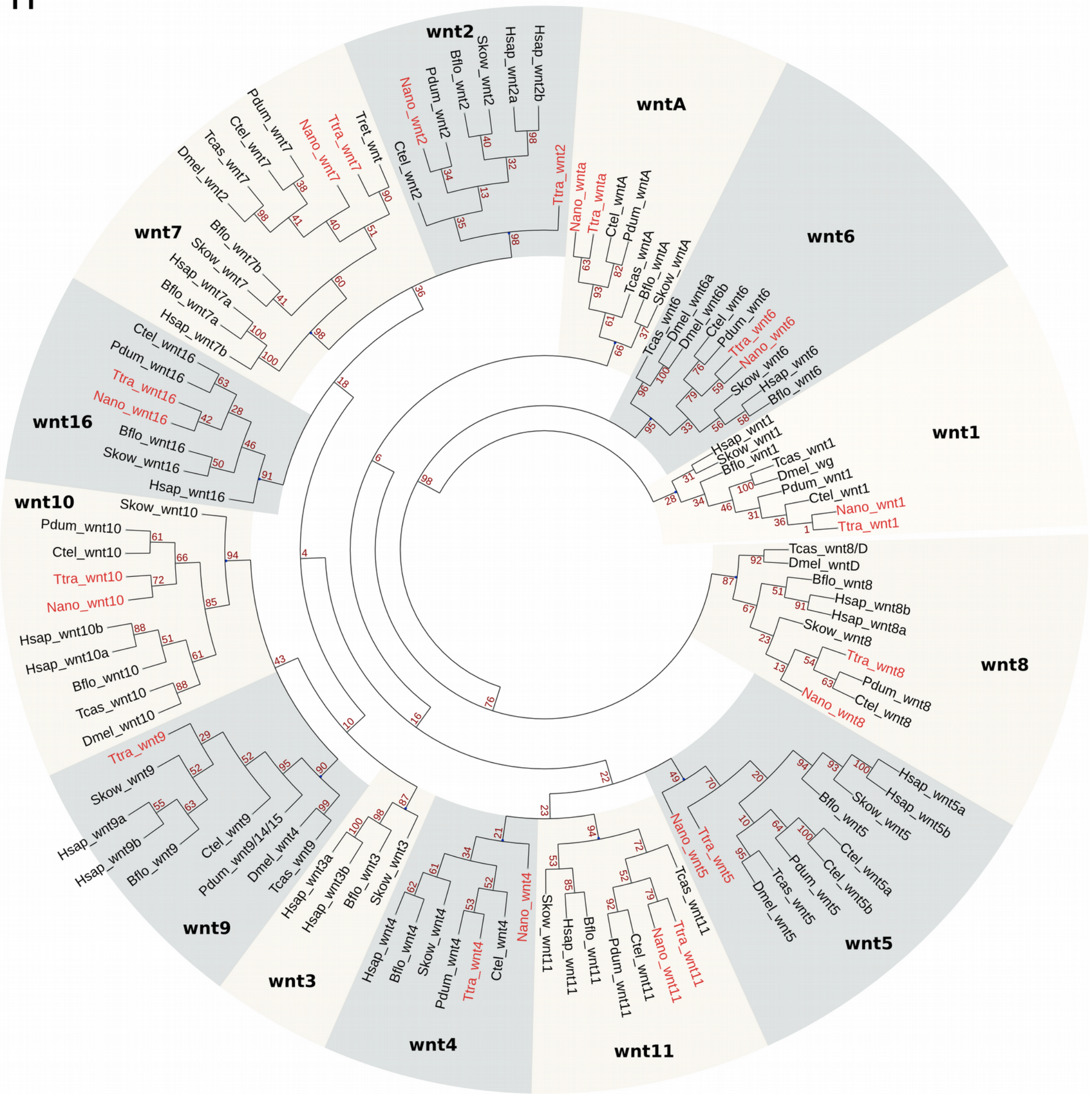

Supplementary Figure S11. Orthology assignment for the brachiopod candidate genes used in this study. (a) *hh*. (b) *gli*. (c) *ptc*. (d) *smo*. (e) *en*. (f) *pax*. (g) *fgf*. (h) *wnt*. Cladograms show branch support values and brachiopod orthologs in red.

Supplementary tables

Supplementary Table S1. Summary of embryonic expression of *en*, *pax6* and *pax2/5/8* across bilaterians. Dashes represent absence of expression and question marks represent missing data. Expression information based on Cephalochordata *en*[3], *pax6*[6] and *pax2/5/8*[7]; Urochordata *en*[8–10], *pax6*[11] and *pax2/5/8*[10,12,13]; Vertebrata *en*[14–22], *pax6*[23–25] and *pax2/5/8*[26–28]; Hemichordata *en*, *pax6* and *pax2/5/8*[29,30]; Echinodermata *en*[31–34] and *pax6*[35]; Nematoda *en*[36], *pax6*[37] and *pax2/5/8*[38]; Tardigrada *en*[39]; Onychophora *en*[5,40], *pax6*[41,42] and *pax2/5/8*[42]; Arthropoda *en*[15,43–56], *pax6*[57] and *pax2/5/8*[58]; Rotifera *pax6*[59]; Platyhelminthes *pax6*[60]; Mollusca *en*[61–66], *pax6*[67–69] and *pax2/5/8*[70,71]; Annelida *en*[1,2,72–75], *pax6*[76–78] and *pax2/5/8*[77]; Nemertea *pax6*[79].

**taxon**	***en* (ectoderm)**	***en* (mesoderm)**	***pax6***	***pax2/5/8***
Cephalochordata	Bilateral domains in the brain.	Transverse stripes at posterior region of first eight somites.	Anterior third of neural plate.	Bilateral clusters at the neural plate around fifth somite.
Urochordata	Anterior neuroectoderm.	-	Animal hemisphere in two bilaterally arranged domains.	Bilateral pair of neural tube cells (coexpressed or intercalated with en).
Vertebrata	Mid/hindbrain boundary.	Posterior portion of somites after morphology (muscle pioneers).	Anterior domain bordering the di/mesencephalon border and developing eyes.	Mid/hindbrain boundary. Pronephros, otic vesicle, endostyle.
Hemichordata	Transverse stripe anterior to collar/trunk boundary.	-	Anterior domain bordering the collar/trunk boundary, overlap with *en* domain.	Posterior expression (trunk) adjacent to *en*.
Echinodermata	Pair of serotonin positive cells around the mouth. Adult nervous system.	Lining coelomic sacs.	Anterior wall of the archenteron in the late gastrula.	?
Chaetognatha	?	?	?	?
Nematoda	Bilateral row of epidermal cells (seam cells) and anterior neurons.	-	Anterior head cells.	Egg-laying system during organogenesis.
Tardigrada	Transverse stripes corresponding to posterior region of underlying endomesodermal pouches before ectodermal segmentation.	-	?	?
Onychophora	Segmental transverse stripes at posterior region of segment with no precise boundaries.	Pair of domains in presomitic mesoderm before segments, but sequentially after morphology.	Cephalic lobe expression posterior of the protocerebral/ocular segment.	Segmental expression matching nephridial openings.
Arthropoda	Segmental transverse stripes at parasegmental boundary and posterior region of segment before	Transient segmental expression.	Forming brain, eye primordia, ventral nerve cord and lateral sense organs.	Few cells per segment.

	morphology. Posterior of imaginal discs, adult abdominal segments and nervous system.			
Scalidophora	?	?	?	?
Rotifera	?	?	Anterior bilateral patches.	?
Micrognathozoa	?	?	?	?
Gnathostomulida	?	?	?	?
Platyhelminthes	?	?	Eyes.	?
Gastrotricha	?	?	?	?
Mollusca	Delimiting shell compartment.	-	Optic region, brain and arms.	Apical pole during embryogenesis, anterior region of the mantle, sensory systems and cephalopod brain.
Annelida	Variable. Segmental expression mainly associated to neurons but can occur before morphology.	Mesodermal derivatives (nephrostome).	Anterior bilateral patches abutting the prototroch and eyes. Nervous system before segmentation.	Trunk nervous system.
Nemertea	?	?	Anterior dorsolateral patches in regenerating eyes.	?
Brachiopoda	Two lateral pairs with anterior domain bordering the apical/mantle boundary.	Posterior portion of two coelomic sacs.	Broad anterior domain bordering the apical/mantle boundary.	Expressed in the mantle lobe.
Phoronida	?	?	?	?
Bryozoa	?	?	?	?

Supplementary Table S2. Gene specific primer pairs used for molecular cloning in *T. transversa* (Ttra) and *N. anomala* (Nano), and gene accession numbers in NCBI.

gene	primers	NCBI accession
Ttra en	R1 CCATCAGATGAAGAGCCAGACCATTTCG	KT253953
	R2 AGTTTTGCTCGTTTGTTTTGGAACC	
Ttra fgf8	F CCGATAGCTTTGGAAGCAAG	KT253954
	R CAATGCCAACAATTCAACCA	
Ttra gli	F CCAGTGAAGCATCTCAAGTGTCG	KT253955
	R CGGTTGCGTGCCATACTATTTTG	
Ttra hh	F AGTGGGAGTTCAGATGGACG	KT253956
	R ATGCTCTCACACGGTCTCAA	
Ttra pax6	F1 GGATAGTGGAACTGGCACACAGCGGAG	KT253957
	F2 CGGATGTGTGAGCAAGATCCTCGGGAG	
Ttra pax258	F TTGCCTGGGAAATACGGGAC	KT253958
	R ATCCAGCTGGTTGACTGGTG	
Ttra ptc	F TTACAGCAGTCAAGAAAGTGGTCG	KT253959
	R TGGGGTTGGATGGATGTTAGC	
Ttra smo	F TGGTCACATTCATTGTCCACCTG	KT253960
	R ATCCTCGGTTTCCTCCTTCTCG	
Ttra wnt1	F TAGCACACACAGGCAAGATAGTCC	KT253961
	R GGAGTAGCAAGTGGAAATGGGG	
Nano en	F ATGTACTCACAGGAGGAGCCACTC	KT253962
	R AGTTGCAGAGCCAATCCGTG	
Nano fgf8	F CAACACGGACACTCCAGAAA	KT253963
	R TATACGGGCTGTTGGTGTCA	
Nano gli	F TCAACAAGGCTACAGTCAGC	KT253964
	R TGCCTTTTATCCTCGTGACG	
Nano hh	F ACCTGAGCAGACGACCAGTT	KT253965
	R AAAATCCCCCAGTTCAAACC	
Nano pax6	F GCAACAGAAGTCAACATGCC	KT253966
	R GACAGGCTGATGGATTGAGG	
Nano pax258	F TGGCTGTGTGAGCAAGATACTCG	KT253967
	R CTGTGAGGAGAGGAGGCATTGTAG	
Nano ptc1	F GGACCCATCCAGCTTTTAGG	KT253968
	R ATCCTGTGTCCAGTCAATGC	
Nano smo	F GCATCCTCTGTACGTGAAGC	KT253969
	R CTCGTACCCAGTGGTTTACG	
Nano wnt1	F CGAGGAACTAAGTGGTGGACATTAG	KT253970
	R TTTCCATCAAGCCCCCTTGG	

References

1. Seaver, E. C. & Kaneshige, L. M. Expression of ’segmentation’ genes during larval and juvenile development in the polychaetes *Capitella* sp. i and *H. elegans*. *Dev. Biol.* **289,** 179–194 (2006).

2. Prud’homme, B. *et al.* Arthropod-like expression patterns of *engrailed* and *wingless* in the annelid *Platynereis dumerilii* suggest a role in segment formation. *Curr. Biol.* **13,** 1876–1881 (2003).

3. Holland, L. Z., Kene, M., Williams, N. A. & Holland, N. D. Sequence and embryonic expression of the amphioxus *engrailed* gene (*AmphiEn*): The metameric pattern of transcription resembles that of its segment-polarity homolog in *drosophila*. *Development* **124,** 1723–1732 (1997).

4. Wedeen, C. J., Kostriken, R. G., Leach, D. & Whitington, P. Segmentally iterated expression of an *engrailed*-class gene in the embryo of an australian onychophoran. *Dev. Genes Evol.* **207,** 282–286 (1997).

5. Eriksson, B. J., Tait, N. N., Budd, G. E. & Akam, M. The involvement of *engrailed* and *wingless* during segmentation in the onychophoran *Euperipatoides kanangrensis* (Peripatopsidae: Onychophora) (Reid 1996). *Dev. Genes Evol.* **219,** 249–264 (2009).

6. Glardon, S., Holland, L. Z., Gehring, W. J. & Holland, N. D. Isolation and developmental expression of the amphioxus *Pax-6* gene (*AmphiPax-6*): Insights into eye and photoreceptor evolution. *Development* **125,** 2701–2710 (1998).

7. Kozmik, Z. *et al.* Characterization of an amphioxus paired box gene, *AmphiPax2/5/8*: Developmental expression patterns in optic support cells, nephridium, thyroid-like structures and pharyngeal gill slits, but not in the midbrain-hindbrain boundary region. *Development* **126,** 1295–1304 (1999).

8. Jiang, D. & Smith, W. C. An ascidian *engrailed* gene. *Dev. Genes Evol.* **212,** 399–402 (2002).

9. Imai, K. S., Satoh, N. & Satou, Y. Region specific gene expressions in the central nervous system of the ascidian embryo. *Mech. Dev.* **119 Suppl 1,** S275–7 (2002).

10. Cañestro, C., Bassham, S. & Postlethwait, J. Development of the central nervous system in the larvacean *Oikopleura dioica* and the evolution of the chordate brain. *Dev. Biol.* **285,** 298–315 (2005).

11. Glardon, S., Callaerts, P., Halder, G. & Gehring, W. J. Conservation of *Pax-6* in a lower chordate, the ascidian *Phallusia mammillata*. *Development* **124,** 817–825 (1997).

12. Wada, H., Saiga, H., Satoh, N. & Holland, P. W. Tripartite organization of the ancestral chordate brain and the antiquity of placodes: Insights from ascidian *Pax-2/5/8*, *Hox* and *Otx* genes. *Development* **125,** 1113–1122 (1998).

13. Bassham, S., Cañestro, C. & Postlethwait, J. H. Evolution of developmental roles of *Pax2/5/8* paralogs after independent duplication in urochordate and vertebrate lineages. *BMC Biol.* **6,** 35 (2008).

14. Njølstad, P. R. & Fjose, A. Insitu hybridization patterns of zebrafish homeobox genes homologous to *hox-2.1* and *en-2* of mouse. *Biochem. Biophys. Res. Commun.* **157,** 426–432 (1988).

15. Patel, N. H. *et al.* Expression of *engrailed* proteins in arthropods, annelids, and chordates. *Cell* **58,** 955–968 (1989).

16. Brivanlou, A. H. & Harland, R. M. Expression of an *engrailed*-related protein is induced in the anterior neural ectoderm of early *Xenopus* embryos. *Development* **106,** 611–617 (1989).

17. Hatta, K., Bremiller, R., Westerfield, M. & Kimmel, C. B. Diversity of expression of *engrailed*-like antigens in zebrafish. *Development* **112,** 821–832 (1991).

18. Davis, C. A., Holmyard, D. P., Millen, K. J. & Joyner, A. L. Examining pattern formation in mouse, chicken and frog embryos with an *En*-specific antiserum. *Development* **111,** 287–298 (1991).

19. Ekker, M., Wegner, J., Akimenko, M. A. & Westerfield, M. Coordinate embryonic expression of three zebrafish *engrailed* genes. *Development* **116,** 1001–1010 (1992).

20. Holland, N. D., Holland, L. Z., Honma, Y. & Fujii, T. *Engrailed* expression during development of a lamprey, *Lampetra japonica*: A possible clue to homologies between agnathan and gnathostome muscles of the mandibular arch. *Dev. Growth Differ.* **35,** 153–160 (1993).

21. Danielian, P. S. & McMahon, A. P. *Engrailed-1* as a target of the Wnt-1 signalling pathway in vertebrate midbrain development. *Nature* **383,** 332–334 (1996).

22. Joyner, A. L. *Engrailed*, *Wnt* and *Pax* genes regulate midbrain-hindbrain development. *Trends Genet.* **12,** 15–20 (1996).

23. Walther, C. & Gruss, P. *Pax-6*, a murine paired box gene, is expressed in the developing CNS. *Development* **113,** 1435–1449 (1991).

24. Püschel, A. W., Gruss, P. & Westerfield, M. Sequence and expression pattern of *pax-6* are highly conserved between zebrafish and mice. *Development* **114,** 643–651 (1992).

25. Derobert, Y., Baratte, B., Lepage, M. & Mazan, S. *Pax6* expression patterns in *Lampetra fluviatilis* and *Scyliorhinus canicula* embryos suggest highly conserved roles in the early regionalization of the vertebrate brain. *Brain Res. Bull.* **57,** 277–280 (2002).

26. Pfeffer, P. L., Gerster, T., Lun, K., Brand, M. & Busslinger, M. Characterization of three novel members of the zebrafish *Pax2/5/8* family: Dependency of *Pax5* and *Pax8* expression on the *Pax2.1* (*noi*) function. *Development* **125,** 3063–3074 (1998).

27. Heller, N. & Brändli, A. W. *Xenopus Pax-2/5/8* orthologues: Novel insights into *Pax* gene evolution and identification of *Pax-8* as the earliest marker for otic and pronephric cell lineages. *Dev. Genet.* **24,** 208–219 (1999).

28. McCauley, D. W. & Bronner-Fraser, M. Conservation of *Pax* gene expression in ectodermal placodes of the lamprey. *Gene* **287,** 129–139 (2002).

29. Lowe, C. J. *et al.* Anteroposterior patterning in hemichordates and the origins of the chordate nervous system. *Cell* **113,** 853–865 (2003).

30. Pani, A. M. *et al.* Ancient deuterostome origins of vertebrate brain signalling centres. *Nature* **483,** 289–294 (2012).

31. Dolecki, G. J. & Humphreys, T. An *engrailed* class homeo box gene in sea urchins. *Gene* **64,** 21–31 (1988).

32. Lowe, C. J. & Wray, G. A. Radical alterations in the roles of homeobox genes during echinoderm evolution. *Nature* **389,** 718–721 (1997).

33. Byrne, M., Cisternas, P. A., Elia, L. & Relf, B. *Engrailed* is expressed in larval development and in the radial nervous system of *Patiriella* sea stars. *Dev. Genes Evol.* **215,** 608–617 (2005).

34. Yaguchi, S., Nakajima, Y., Wang, D. & Burke, R. D. Embryonic expression of *engrailed* in sea urchins. *Gene Expr. Patterns* **6,** 566–571 (2006).

35. Omori, A., Akasaka, K., Kurokawa, D. & Amemiya, S. Gene expression analysis of *Six3*, *Pax6*, and *Otx* in the early development of the stalked crinoid *Metacrinus rotundus*. *Gene Expr. Patterns* **11,** 48–56 (2011).

36. Cassata, G. *et al. Ceh-16/engrailed* patterns the embryonic epidermis of *Caenorhabditis elegans*. *Development* **132,** 739–749 (2005).

37. Chisholm, A. D. & Horvitz, H. R. Patterning of the *Caenorhabditis elegans* head region by the *Pax-6* family member *vab-3*. *Nature* **377,** 52–55 (1995).

38. Rajakumar, V. & Chamberlin, H. M. The *Pax2/5/8* gene *egl-38* coordinates organogenesis of the *C. elegans* egg-laying system. *Dev. Biol.* **301,** 240–253 (2007).

39. Gabriel, W. N. & Goldstein, B. Segmental expression of *Pax3/7* and *engrailed* homologs in tardigrade development. *Dev. Genes Evol.* **217,** 421–433 (2007).

40. Franke, F. A. & Mayer, G. Controversies surrounding segments and parasegments in onychophora: Insights from the expression patterns of four ‘segment polarity genes’ in the peripatopsid *Euperipatoides rowelli*. *PLoS One* **9,** e114383 (2014).

41. Eriksson, B. J., Samadi, L. & Schmid, A. The expression pattern of the genes *engrailed, pax6, otd* and *six3* with special respect to head and eye development in *Euperipatoides kanangrensis* Reid 1996 (Onychophora: Peripatopsidae). *Dev. Genes Evol.* **223,** 237–246 (2013).

42. Franke, F. A., Schumann, I., Hering, L. & Mayer, G. Phylogenetic analysis and expression patterns of *Pax* genes in the onychophoran *Euperipatoides rowelli* reveal a novel bilaterian *P*ax subfamily. *Evol. Dev.* **17,** 3–20 (2015).

43. Fjose, A., McGinnis, W. J. & Gehring, W. J. Isolation of a homoeo box-containing gene from the *engrailed* region of *Drosophila* and the spatial distribution of its transcripts. *Nature* **313,** 284–289 (1985).

44. Kornberg, T., Sidén, I., O’Farrell, P. & Simon, M. The *engrailed* locus of *Drosophila*: In situ localization of transcripts reveals compartment-specific expression. *Cell* **40,** 45–53 (1985).

45. DiNardo, S. & O'Farrell, P. H. Establishment and refinement of segmental pattern in the *Drosophila* embryo: Spatial control of *engrailed* expression by pair-rule genes. *Genes Dev.* **1,** 1212–1225 (1987).

46. Fleig, R. *Engrailed* expression and body segmentation in the honeybee *Apis mellifera*. *Rouxs. Arch. Dev. Biol.* **198,** 467–473 (1990).

47. Whitington, P. M., Meier, T. & King, P. Segmentation, neurogenesis and formation of early axonal pathways in the centipede, *Ethmostigmus rubripes* (Brandt). *Rouxs. Arch. Dev. Biol.* **199,** 349–363 (1991).

48. Dougan, S. & DiNardo, S. *Drosophila wingless* generates cell type diversity among *engrailed* expressing cells. *Nature* **360,** 347–350 (1992).

49. Manzanares, M., Marco, R. & Garesse, R. Genomic organization and developmental pattern of expression of the *engrailed* gene from the brine shrimp *Artemia*. *Development* **118,** 1209–1219 (1993).

50. Brown, S. J., Patel, N. H. & Denell, R. E. Embryonic expression of the single *Tribolium engrailed* homolog. *Dev. Genet.* **15,** 7–18 (1994).

51. Manzanares, M., Williams, T. A., Marco, R. & Garesse, R. Segmentation in the crustacean *Artemia*: *Engrailed* staining studied with an antibody raised against the *Artemia* protein. *Rouxs. Arch. Dev. Biol.* **205,** 424–431 (1996).

52. Ahzhanov, A. & Kaufman, T. C. Evolution of distinct expression patterns for *engrailed* paralogues in higher crustaceans (Malacostraca). *Dev. Genes Evol.* **210,** 493–506 (2000).

53. Marie, B. & Bacon, J. P. Two *engrailed*-related genes in the cockroach: Cloning, phylogenetic analysis, expression and isolation of splice variants. *Dev. Genes Evol.* **210,** 436–448 (2000).

54. Damen, W. G. M. Parasegmental organization of the spider embryo implies that the parasegment is an evolutionary conserved entity in arthropod embryogenesis. *Development* **129,** 1239–1250 (2002).

55. Mellenthin, K. *et al.* Wingless signaling in a large insect, the blowfly *Lucilia sericata*: A beautiful example of evolutionary developmental biology. *Dev. Dyn.* **235,** 347–360 (2006).

56. Farzana, L. & Brown, S. J. Hedgehog signaling pathway function conserved in *Tribolium* segmentation. *Dev. Genes Evol.* **218,** 181–192 (2008).

57. Quiring, R., Walldorf, U., Kloter, U. & Gehring, W. J. Homology of the *eyeless* gene of *Drosophila* to the *Small eye* gene in mice and *Aniridia* in humans. *Science* **265,** 785–789 (1994).

58. Czerny, T., Bouchard, M., Kozmik, Z. & Busslinger, M. The characterization of novel *Pax* genes of the sea urchin and *Drosophila* reveal an ancient evolutionary origin of the *Pax2/5/8* subfamily. *Mech. Dev.* **67,** 179–192 (1997).

59. Boell, L. A. & Bucher, G. Whole-mount in situ hybridization in the rotifer *Brachionus plicatilis* representing a basal branch of lophotrochozoans. *Dev. Genes Evol.* **218,** 445–451 (2008).

60. Callaerts, P. *et al.* Isolation and expression of a *Pax-6* gene in the regenerating and intact Planarian *Dugesia(G)tigrina*. *Proceedings of the National Academy of Sciences* **96,** 558–563 (1999).

61. Moshel, S. M., Levine, M. & Collier, J. R. Shell differentiation and *engrailed* expression in the *Ilyanassa* embryo. *Dev. Genes Evol.* **208,** 135–141 (1998).

62. Jacobs, D. K. *et al.* Molluscan *engrailed* expression, serial organization, and shell evolution. *Evol. Dev.* **2,** 340–347 (2000).

63. Wanninger, A. & Haszprunar, G. The expression of an engrailed protein during embryonic shell formation of the tusk-shell, *Antalis entalis* (mollusca, scaphopoda). *Evolution and Development* **3,** 312–321 (2001).

64. Nederbragt, A. J., Loon, A. E. van & Dictus, W. J. A. G. Expression of *Patella vulgata* orthologs of *engrailed* and *dpp-bMP2/4* in adjacent domains during molluscan shell development suggests a conserved compartment boundary mechanism. *Dev. Biol.* **246,** 341–355 (2002).

65. Baratte, S., Andouche, A. & Bonnaud, L. *Engrailed* in cephalopods: A key gene related to the emergence of morphological novelties. *Dev. Genes Evol.* **217,** 353–362 (2007).

66. Hohagen, J., Herlitze, I. & Jackson, D. J. An optimised whole mount in situ hybridisation protocol for the mollusc *Lymnaea stagnalis*. *BMC Dev. Biol.* **15,** 19 (2015).

67. Tomarev, S. I. *et al.* Squid Pax-6 and eye development. *Proc. Natl. Acad. Sci. U. S. A.* **94,** 2421–2426 (1997).

68. Hartmann, B. *et al. Pax6* in the sepiolid squid *Euprymna scolopes*: Evidence for a role in eye, sensory organ and brain development. *Mech. Dev.* **120,** 177–183 (2003).

69. Navet, S., Andouche, A., Baratte, S. & Bonnaud, L. *Shh* and *Pax6* have unconventional expression patterns in embryonic morphogenesis in *Sepia officinalis* (Cephalopoda). *Gene Expr. Patterns* **9,** 461–467 (2009).

70. O'Brien, E. K. & Degnan, B. M. Expression of *Pax258* in the gastropod statocyst: Insights into the antiquity of metazoan geosensory organs. *Evol. Dev.* **5,** 572–578 (2003).

71. Wollesen, T., Rodríguez Monje, S. V., Todt, C., Degnan, B. M. & Wanninger, A. Ancestral role of *pax2/5/8* in molluscan brain and multimodal sensory system development. *BMC Evol. Biol.* **15,** 231 (2015).

72. Wedeen, C. J. & Weisblat, D. A. Segmental expression of an *engrailed*-class gene during early development and neurogenesis in an annelid. *Development* **113,** 805–814 (1991).

73. Lans, D., Wedeen, C. J. & Weisblat, D. A. Cell lineage analysis of the expression of an *engrailed* homolog in leech embryos. *Development* **117,** 857–871 (1993).

74. Bely, A. E. & Wray, G. A. Evolution of regeneration and fission in annelids: Insights from *engrailed*- and *orthodenticle*-class gene expression. *Development* **128,** 2781–2791 (2001).

75. Seaver, E. C., Paulson, D. A., Irvine, S. Q. & Martindale, M. Q. The spatial and temporal expression of *Ch-en*, the *engrailed* gene in the polychaete *Chaetopterus*, does not support a role in body axis segmentation. *Dev. Biol.* **236,** 195–209 (2001).

76. Arendt, D., Tessmar, K., Campos-Baptista, M.-I. M. de, Dorresteijn, A. & Wittbrodt, J. Development of pigment-cup eyes in the polychaete *Platynereis dumerilii* and evolutionary conservation of larval eyes in Bilateria. *Development* **129,** 1143–1154 (2002).

77. Denes, A. S. *et al.* Molecular architecture of annelid nerve cord supports common origin of nervous system centralization in Bilateria. *Cell* **129,** 277–288 (2007).

78. Quigley, I. K., Xie, X. & Shankland, M. *Hau-Pax6A* expression in the central nervous system of the leech embryo. *Dev. Genes Evol.* **217,** 459–468 (2007).

79. Tarpin, M., Gehring, W. J. & Bièrne, J. Reverse homeosis in homeotically reconstructed ribbonworms. *Proc. Natl. Acad. Sci. U. S. A.* **96,** 11900–11903 (1999).
